# Medical students benefit from the use of ultrasound when learning peripheral IV techniques

**DOI:** 10.1186/2036-7902-4-2

**Published:** 2012-03-06

**Authors:** Scott R Osborn, Joelle Borhart, Michael S Antonis

**Affiliations:** 1 Southside Regional Medical Center, 200 Medical Park Blvd., Petersburg, VA, 23805, USA; 2 MedStar Georgetown University Hospital/Washington Hospital Center Department of Emergency Medicine, 110 Irving Street, NW Washington, DC, 20010, USA; 3 MedStar Emergency Medicine Ultrasound and Fellowship, Georgetown University Hospital/Washington Hospital Center Emergency Medicine Residency Program, 110 Irving Street, NW Washington, DC, 20010, USA; 4 Virginia Mason Medical Center, 1100 9th Avenue, Seattle, WA, 98101, USA

**Keywords:** ultrasonography, teaching, catheterization, intravenous

## Abstract

**Background:**

Recent studies support high success rates after a short learning period of ultrasound IV technique, and increased patient and provider satisfaction when using ultrasound as an adjunct to peripheral IV placement. No study to date has addressed the efficacy for instructing ultrasound-naive providers. We studied the introduction of ultrasound to the teaching technique of peripheral IV insertion on first- and second-year medical students.

**Methods:**

This was a prospective, randomized, and controlled trial. A total of 69 medical students were randomly assigned to the control group with a classic, landmark-based approach (*n *= 36) or the real-time ultrasound-guided group (*n *= 33). Both groups observed a 20-min tutorial on IV placement using both techniques and then attempted vein cannulation. Students were given a survey to report their results and observations by a 10-cm visual analog scale. The survey response rate was 100%.

**Results:**

In the two groups, 73.9% stated that they attempted an IV previously, and 63.7% of students had used an ultrasound machine prior to the study. None had used ultrasound for IV access prior to our session. The average number of attempts at cannulation was 1.42 in either group. There was no difference between the control and ultrasound groups in terms of number of attempts (*p *= 0.31). In both groups, 66.7% of learners were able to cannulate in one attempt, 21.7% in two attempts, and 11.6% in three attempts. The study group commented that they felt they gained more knowledge from the experience (*p *< 0.005) and that it was easier with ultrasound guidance (*p *< 0.005).

**Conclusion:**

Medical students feel they learn more when using ultrasound after a 20-min tutorial to place IVs and cannulation of the vein feels easier. Success rates are comparable between the traditional and ultrasound teaching approaches.

## Introduction

Obtaining peripheral intravenous [PIV] access is a basic medical skill. Medical students traditionally acquire PIV knowledge on rotations or during a brief, highly variable and institution-dependent, practicum. PIV placement is described as one of the fourteen learning objectives required by the International Federation for Emergency Medicine [EM] Undergraduate EM curriculum. PIV access plays a crucial role at the center of patient care.

Studies indicate that use of real-time ultrasound [US] visualization for PIV placement lessens the need for central access and increases rates of successful cannulation [1]. However, no study has yet addressed the benefit of using US early in teaching naive medical students the skill of PIV placement. Use of US by new learners has transformed central line placement, and the question remains if similar results could be expected of their peripheral counterparts. We studied whether the adjunct use of US instruction for PIV techniques improved learner cannulation success rates and learner satisfaction with US guidance versus the traditional landmark technique.

## Methodology

This was a prospective, randomized, and controlled trial with a convenience sample of students. Willing first- and second-year medical students (*n *= 69) were recruited by email and were randomly assigned to either the landmark-only control (*n *= 36) group or the ultrasound study (*n *= 33) group. Our institutional review board approved the study protocol, and we obtained a signed, informed consent from each study participant.

The study was conducted from 1 September 2008 to 31 October 2008, at Georgetown University and Medical Center. The PIV catheters used were 20-gauge, 1-in., shielded IV catheters. Control group participants observed a 20-min scripted tutorial on PIV placement and attempted cannulation on other medical students. Study group participants observed a 20-min scripted tutorial on PIV placement using the SonoSite MicroMaxx ultrasound machine (Bothell, WA, USA), using the vascular setting and linear probe. The technique utilized was the cross-sectional (short-axis) approach with two-handed, real-time visualization on other medical students.

Successful venous cannulation was defined as blood return via a 5-cc syringe from the inserted angiocatheter with removal of the needle. The participants were instructed to remove the catheter after the instructors confirmed venous blood return. No practice attempts were afforded to either group, and each attempt was documented until success was achieved. Surveys were given to the students on successful completion of the cannulation of the vein.

Survey information was obtained on a single sheet of paper using a 10-cm visual analog scale (Appendix). There was a 100% survey response rate.

## Results

**Figure 1:**
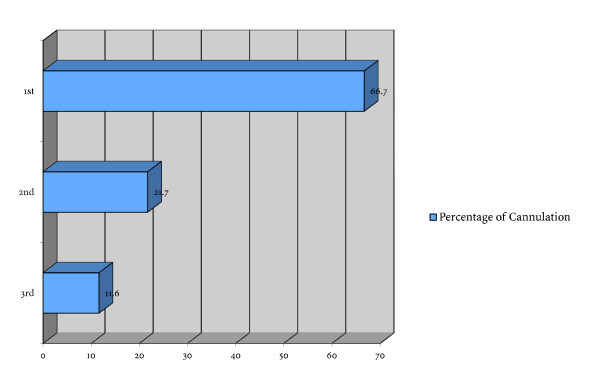
**Percentage of successful cannulation with number of attempts**.

There was no difference between groups in regards to previous US or IV placement experience. The average number of attempts per cannulation was 1.36 (95% CI 1.07 to 1.65) in the ultrasound group and 1.38 (95% CI 1.08 to 1.68) in the control group (*p *= 0.31). The ultrasound group scored the experience easier than the control group (mean 5.91 vs. 3.65, *p *< 0.01). The ultrasound group responded that they gained more knowledge of the mechanics of placing an IV than the control group (6.55 vs. 5.00, *p *< 0.01) using the visual analog scale with 1 being difficult or less knowledge and 10 being considered easy or gaining more knowledge of the mechanics (Table 1). Every student was able to successfully cannulate a vein by the third attempt with the overwhelming majority placing an IV in two attempts (Figure 1).

**Table 1:** Survey results

Questionnaire results	Variable	Control	Ultrasound	*P *value
Group size	(*n *= X)	36	33	
Prior US experience?	Yes	24	20	0.69
Prior IV attempts?	Yes	29	22	0.40
How many attempts did it take to cannulate a vein?	Mean	1.38	1.36	0.30
How easy was the cannulation? (visual-analog scale 1-10)	Mean	3.66	5.91	< 0.001
How much knowledge of IV placement do you feel you gained from the experience?	Mean	5.00	6.55	< 0.001

## Discussion

Obtaining PIV access is an important medical skill set that often is neglected in medical student education in part because PIV placement has been traditionally thought of as a nurse's or tech's duty. However, the job of 'getting access' is increasingly becoming the responsibility of the physician, especially in patients with historically difficult peripheral veins to cannulate (i.e., past or current IV drug users, patients with end-stage renal disease, morbidly obese, and edematous patients) [2,3].

The ability to gain venous access without a central line affords the physician a less time-intensive and invasive procedure with fewer known complications. The fiscal reality for hospital systems that payors will no longer reimburse for Central Line-Associated BloodStream Infections (CLABSI) also lends credence to the claim that the best central line is the one not placed from an infectious disease standpoint [4].

The emergence of ultrasound in medical school training is becoming more prevalent with the introduction of real-time US in conjunction with first-year medical student anatomy classes [5]. The system-based approach for medical education allows the introduction of different modes of imaging specific for each anatomical system. Ultrasound provides a vehicle for education free of ionizing radiation or limitation by the expense of an MRI scanner for real-time imaging and instructor-based feedback. We believe that it is clinically beneficial for medical students to be exposed to formal, organized teaching sessions on PIV placement as part of their preclinical medical education with increased exposure to ultrasound.

## Conclusion

We anticipated that the number of attempts would be less with ultrasound, but there was no difference suggesting that psychomotor skills required with ultrasound need to be developed over time. Although testing the students on a 'difficult access' patient was not done in this study, the applicability of US as an adjunct to PIV placement is well documented. Preclinical medical student teaching with ultrasound is the next step and is demonstrated in our study by the improved understanding of the mechanics of PIV insertion by medical students.

## Competing interests

The authors declare that they have no competing interests.

## Authors' contributions

SRO conceived and designed the study with consultation by MSA. SRO and MSA drafted and revised the manuscript, figures, and tables. SRO, JB, and MSA collected the data and coordinated the study. SRO performed the statistical analysis. All authors read and approved the final manuscript.

## Appendix

### Medical student peripheral IV questionaire

Please answer each of the questions below.

**Have you used ultrasound before? **________

**Have you attempted an I.V. before? **________

For each of the following questions, please make one vertical line between the brackets below indicating your response.

Did you find it easier to place an I.V. using ultrasound?

Much harder [          |          ] Much easier

Do you think that using ultrasound to place a peripheral I.V. has provided you with more knowledge of the skill than the basic landmark technique alone?

Much less [          |          ] Much more
